# Prevalence of Vestibular Disorder in Older People Who Experience Dizziness

**DOI:** 10.3389/fneur.2015.00268

**Published:** 2015-12-24

**Authors:** Allan T. Chau, Jasmine C. Menant, Patrick P. Hübner, Stephen R. Lord, Americo A. Migliaccio

**Affiliations:** ^1^Neuroscience Research Australia, University of New South Wales, Sydney, NSW, Australia; ^2^Department of Otolaryngology – Head and Neck Surgery, Johns Hopkins University School of Medicine, Baltimore, MD, USA

**Keywords:** aged, postural balance, vestibular function tests, benign paroxysmal positional vertigo, peripheral and central vestibular function

## Abstract

Dizziness and imbalance are clinically poorly defined terms, which affect ~30% of people over 65 years of age. In these people, it is often difficult to define the primary cause of dizziness, as it can stem from cardiovascular, vestibular, psychological, and neuromuscular causes. However, identification of the primary cause is vital in determining the most effective treatment strategy for a patient. Our aim is to accurately identify the prevalence of benign paroxysmal positional vertigo (BPPV), peripheral, and central vestibular hypofunction in people aged over 50 years who had experienced dizziness within the past year. Seventy-six participants aged 51–92 (mean ± SD = 69 ± 9.5 years) were tested using the head thrust dynamic visual acuity (htDVA) test, dizziness handicap inventory (DHI), as well as sinusoidal and unidirectional rotational chair testing, in order to obtain data for htDVA score, DHI score, sinusoidal (whole-body, 0.1–2 Hz with peak velocity at 30°/s) vestibulo-ocular reflex (VOR) gain and phase, transient (whole-body, acceleration at 150°/s^2^ to a constant velocity rotation of 50°/s) VOR gain and time constant (TC), optokinetic nystagmus (OKN) gain, and TC (whole-body, constant velocity rotation at 50°/s). We found that BPPV, peripheral and central vestibular hypofunction were present in 38 and 1% of participants, respectively, suggesting a likely vestibular cause of dizziness in these people. Of those with a likely vestibular cause, 63% had BPPV; a figure higher than previously reported in dizziness clinics of ~25%. Our results indicate that htDVA, sinusoidal (particularly 0.5–1 Hz), and transient VOR testing were the most effective at detecting people with BPPV or vestibular hypofunction, whereas DHI and OKN were effective at only detecting non-BPPV vestibular hypofunction.

## Introduction

The vestibular system detects and initiates responses to changes in sensations of equilibrium. Disorder in any part of this system, typically occurring due to aging or injury, results in dizziness and imbalance, which contributes to an increased risk of falls (1). Injury to the vestibular system due to trauma, disease, or ototoxic drugs is often localized, e.g., to the peripheral component, whereas injury through aging is thought to affect the vestibular system as a whole. While structural changes within the vestibular system as a whole have been observed due to aging, associations between these changes and dizziness (visual stability), imbalance, gait disturbances, and falls has yet to be established (2), and it is not clear whether the prevalence of dizziness and imbalance in the elderly of ~30% (3) is primarily due to psychological, cardiovascular, muscular, or vestibular system degeneration.

One component of the vestibular system is the angular vestibulo-ocular reflex (VOR), which is responsible for stable vision during head motion by rotating the eyes in the opposite direction to head rotation to maintain gaze and images stationary on the retina. The cristae ampullaris of the semicircular canals, which sense angular head rotations, displays the most profound degeneration in the vestibular end organ, where there is a 40% decrease in hair cells for all canals by age 80 (4–6). Cristae type I hair cell loss with advancing age occurs at twice the rate of the macule, whereas for type II hair cells, it is at the same rate for all five sense organs – the decline is roughly linear with age for both types (5, 6). However, previous studies examining the effect of aging on the angular VOR have been conflicting in their findings. Upon stimulation of the VOR via sinusoidal rotation testing, gain values (gain = eye velocity/head velocity; the ideal gain of the VOR during typical viewing is equal to unity) were shown to decrease with age, whereas phase shift (the difference in time between ideal eye velocity for head movement compensation and actual eye velocity) (7) has generally been demonstrated to increase with age. However, the testing conditions under which these changes were observed were not the same, with differences being attributed to lower (<0.4 Hz) frequency stimuli (8–10) or higher (>2 Hz) frequency stimuli (11, 12). Age-related changes in the dominant time constant (TC) (time taken for eye velocity to decay to 63% of its peak velocity under constant velocity head rotation with unidirectional rotation testing of the VOR) have also been studied, again with conflicting findings. Some studies reporting decreasing TCs (from a mean of 15.1 s to a mean of 11.7 s) with age (12), while others report shorter TCs in younger participants (13), whereas others report no age-related changes to the physiological function of these three (gain, phase, and TC) parameters (14). Therefore, for those within the healthy population who do not experience balance problems, it is unclear whether the anatomical changes described in the literature relate functionally to older people. Research utilizing head thrust dynamic visual acuity (htDVA) has also suggested a link between htDVA scores and propensity to falls within older, community-dwelling populations (15). The rationale behind passive htDVA is that people with vestibular organ injury have problems stabilizing images as only the VOR can keep up with fast head movements, resulting in a decrease in visual acuity during head thrusts [i.e., a difference between static and dynamic visual acuity (DVA)] (16). However, while htDVA is a very specific test (90% unilateral peripheral hypofunction, 90% bilateral hypofunction), its sensitivity in detecting peripheral vestibular hypofunction is limited (23% unilateral peripheral hypofunction, 55% bilateral peripheral hypofunction), meaning that although a bad DVA score indicates a poor VOR, a good score may not always mean a well-functioning VOR (17).

Benign paroxysmal positional vertigo (BPPV) is a condition where the otoconia are dislodged from their usual position within the utricle and migrate into one of the semicircular canals (the posterior canal is most commonly affected due to its anatomical position). When the head is re-oriented relative to gravity, the gravity-dependent movement of the heavier otoconial debris within the affected semicircular canal causes pathological endolymph displacement and a resultant sensation of vertigo. BPPV is the most common form of positional vertigo, accounting for nearly one half of patients with peripheral vestibular disorder. Approximately 18% of people seen in dizziness clinics (18) and 25% of people sent for vestibular testing have BPPV (19). In a population-based survey of 1003 people, the prevalence of BPPV was 2.4% and the 1-year prevalence of BPPV increased with age such that it was seven times higher in those aged 60 years and older compared to those aged 18–39 years (20).

Additionally, research into the efficacy of treatments for dizziness, such as vestibular rehabilitation, has seemingly indicated benefit and improvements in the quality of life of patients suffering from dizziness and imbalance (21). Identification of these people is important so that they can receive rehabilitation treatment. Our aim is to identify accurately the prevalence of BPPV, peripheral and central vestibular hypofunction within people over the age of 50 who had experienced an episode of dizziness, self-reported or documented, within the last year. We sought a snapshot of this particular population because these were the people most likely to present to a physician with dizziness, while acknowledging that this approach would likely result in an over-estimate of the true numbers of people with vestibular disorders in this overall age group. We used the Dizziness Handicap Inventory Questionnaire, Dix–Hallpike test, clinical head impulse test (cHIT), htDVA test, as well as sinusoidal and unidirectional rotational chair testing in order to, respectively, obtain data for BPPV, presence of refixation saccades, DVA, sinusoidal VOR gain and phase (between 0.1 to 2 Hz), transient VOR gain and TC, and optokinetic nystagmus (OKN) gain and TC.

## Materials and Methods

### Participants

We studied 76 people with self-reported dizziness who had experienced at least one dizziness episode within the past year; 44 females and 28 males, aged 51–92 years old (mean = 69 ± 9.5). Participants were recruited through advertisements and mail flyers, the Neuroscience Research Australia website and newsletter, existing clinical networks and by contacting residents of retirement villages. The inclusion criteria were aged 50 years and over, dizziness (self-reported or documented) not currently being treated, living independently in the community or retirement village and able to understand English. The exclusion criteria were presence of a degenerative neurological condition or cognitive impairment. Participants identified on assessment with conditions that required urgent treatment defined as suspected stroke, transient ischemic attack, or other undiagnosed neurological or acute cardiovascular condition, severe depressive symptoms, or severe anxiety symptoms were also excluded from the study and referred for appropriate treatment. No participant included had a history of hypotension, bradycardia, fainting, seizures, or vomiting/migraines due to motion sickness. All participants gave written and informed consent prior to participating in the study. The experimental protocol was approved by the Human Research Ethics Committee at the University of New South Wales.

### Testing Protocol

Baseline testing took 1 day to complete for each participant. No participant was treated on the same day as their testing. Participants completed the Jacobson and Newman dizziness handicap inventory (DHI) (22), followed by the tests below in the order presented.

#### Dix–Hallpike Test to Detect BPPV

Participants underwent the Dix–Hallpike test for vertical canal BPPV using Frenzel lens goggles with integrated video camera for eye image recording (23). With the head turned 45° to one side and extended about 20° backward, the participant was brought from a sitting position to a supine position. Once supine, the eyes were observed for 30 s. If no nystagmus ensued, the participant was brought back to the sitting position and kept stationary for 30 s, after which the other side was tested. Horizontal canal BPPV testing began with the body supine and the head inclined forward 30°. The head was then turned to either side, i.e., the supine roll test. The Epley or particle repositioning maneuver was *not* performed on participants to treat their BPPV during this baseline testing session.

#### Clinical Head Impulse Test

A head impulse consists of a unilateral transient head rotation with peak amplitude ~10°, peak velocity ~150°/s, and peak acceleration ~3000°/s^2^ (16). Passive, five leftward and five rightward, head impulses were manually delivered while the subject fixated the examiner’s nose. The examiner noted whether or not refixation saccades occurred, i.e., positive for cHIT, for each direction of rotation.

#### Dynamic Visual Acuity

Participants who normally wore glasses or contact lenses for distant viewing were instructed to wear them during all DVA tests. Participants were seated 2 m directly in front of a high-resolution (1920 × 1080) 18.1-viewable-inch monitor with a refresh rate of 85 Hz. Static visual acuity was measured first by repeatedly displaying a single optotype (the letter E, randomly rotated each trial by 0°, 90°, 180°, or 270°) on a computer monitor. Participants viewed five optotypes per acuity level (letter size), which decreased in visual acuity levels of 0.1 LogMAR (MAR, minimum angle resolved). The lower the LogMAR score, the better the visual acuity, with LogMAR = −0.3, 0, 0.3, 0.7, 1.0, and 1.3 corresponding to Snellen visual acuity of 20/10, 20/20, 20/40, 20/100, 20/200, and 20/400, respectively. Static visual acuity was determined when the participant failed to correctly identify five optotypes on an acuity level or reached the LogMAR score of 0.000 (Snellen equivalent of 20/20 acuity).

For the dynamic component of the test, two IDG500/ADXL33 IMU (2D gyroscopes) Analog Combo Boards, aligned in the right anterior and left posterior (RALP) and left anterior and right posterior (LARP) vestibular semicircular canal (SCC) planes attached to an adjustable head mount were positioned on the participants head to measure angular head velocity in the horizontal, LARP, and RALP planes. A head thrust (also known as a head impulse) is a manually delivered, unpredictable, unidirectional, rapid head rotation with peak amplitude ~10°, peak velocity ~150°/s, and peak acceleration ~3000°/s^2^. Horizontal head thrusts to assess horizontal canal function were performed first, followed by RALP, and then LARP head thrusts. Each semicircular canal was assessed separately, with head thrusts delivered only in its particular orientation until DVA was determined (i.e., leftward head thrusts tested the left horizontal canal and the DVA score was determined, followed by right horizontal canal testing, and so on). One practice trial for a head thrust was performed before commencing dynamic head thrust DVA (htDVA) testing in each of the planes of the horizontal, superior, and posterior SCCs. During each head thrust, the optotype “E” randomly oriented in one of the four directions (
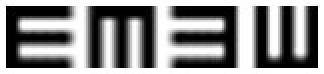
) was displayed on the monitor 2 m in front of the participant when head velocity, sensed by the IDG500/ADXL33 Analog Combo Board, was between 120 and 180°/s for more than 40 ms. The optotype was displayed on the monitor for no longer than 85 ms, during which time the head would have rotated 9°–13.5°. To account for loss of concentration or blinking, the participant was allowed to view each optotype a maximum of three times, at which point the participant was required to guess the orientation. Once the participant indicated a response, the next trial was started, with the test being terminated when the participant incorrectly identified five optotypes at one acuity level or the participant reached the LogMAR score of 0.000. The htDVA test score was calculated by subtracting the static visual acuity LogMAR score from the DVA LogMAR score. As an aid to data interpretation only, we classified the scores as normal [≤0.158, i.e., ≤2 SDs from normal mean, see Ref. (17)], borderline (>0.158 and ≤0.316, 2–4 SDs), and abnormal (>0.316).

#### Rotary Chair Testing

The movements of both eyes were recorded in two dimensions (horizontal and vertical) using video-oculography. Eye position was recorded at 30 fps with a small, light-weight, high speed video camera mounted onto a light-weight scuba mask with an adjustable rubber strap that sat tightly on the bridge of the nose and around the eye sockets to minimize movement of the camera relative to the head. Two infrared light-emitting diodes (LEDs) were directed toward the eyes. The image of the eye was reflected from a hot (infrared) mirror onto the camera, the camera and hot mirror were mounted rigidly onto the mask. Horizontal and vertical eye movements were calibrated *in vivo* by asking the participant to fixate sequentially on the center and then four tips of a cross projected from a mask-mounted cross-hair projector. Eye position was calculated by tracking the pupils in the video images.

The rotary chair consisted of a reinforced car seat with foot support, four point safety harness, and head holder brace, used to tightly secure the participant’s head, torso, and feet to the chair, respectively. The chair sat on a freely rotating hollow shaft attached via gears and a tension controlled toothed-belt to a floor-mounted motor (Baldor SD55-15A1 brushed DC servo motor, USA). The hollow shaft had a position encoder (S2 Optical Kit, USA) to monitor chair position, and slip-ring so that electrical signals, including power supply, could connect with equipment attached to the chair. For example, signals to and from the chair and video data acquisition laptop computer and angular velocity (rate) sensor (GyroPAK3, USA) that measured chair velocity. Chair position and velocity were precisely controlled using a motion controller (NI PCI-7342, USA) and LabVIEW program (National Instruments, USA) running on the control PC located outside of the testing room. Head (angular chair position and velocity) and eye (video-oculography, horizontal and vertical angular eye position, and velocity) data were synchronously sampled at 30 Hz by the acquisition laptop computer (DAQ NI-USB-6008, USA), which wirelessly transmitted these data to the control PC.

The VOR testing protocol consisted of four steps.

1. Each participant was examined for spontaneous nystagmus by asking them to fixate on a point (170 cm) directly in front of them at eye level, and to hold that same gaze position even after the lights were turned out and they were in complete darkness.
2. The visual vestibulo-ocular reflex (VVOR) was tested by sinusoidal horizontal whole-body rotations at a series of frequencies (0.1, 0.2, 0.4, 0.5, 0.8, 1, 1.6, and 2 Hz, all with peak velocity 30°/s). The participant was tested in light, with instructions to visually fixate on a room-fixed point (170 cm) directly in front of them at eye level.
3. The VOR was measured using the VVOR rotation protocol above, the only difference being that the participant was tested in complete darkness.
4. The horizontal VOR was tested during transient rotations (i.e., acceleration steps). From stationary, the chair (and participant) accelerated (~150°/s^2^) up to a constant velocity of 50°/s lasting for 3 min and then de-accelerated (~150°/s^2^) to stationary, remaining so for at least 1 min. Testing was in complete darkness, except for a 1 min period starting precisely 1 min after acceleration commenced. During this 1 min period, OKN was induced by optokinetic stimuli painted on the walls of the room (40° subtending black stripes to 8° subtending white stripes). Participants were told not to fixate on the stripes as they entered their view. The OKN gain and TC were measured using the data collected during this 1 min period in light, whereas the VOR gains and TCs were measured during the 1 min periods in complete darkness immediately post acceleration and de-acceleration. Responses were measured for both leftward and rightward transient rotations.

### Participant Vestibular Rehabilitation Category

Each participant was assessed at a case conference that included a geriatrician, vestibular physiotherapist, applied neurologist, vestibular scientist, and psychologist. Each specialist evaluated the participant’s medical history and performance on a range of depression and anxiety questionnaires as well as sensorimotor, balance, cardiovascular (data not presented here), and vestibular tests, including the tests outlined in this study. Each participant was categorized as having symptoms of dizziness due to vestibular hypofunction making them suitable for vestibular rehabilitation exercises (“Lesion” group), BPPV making them suitable for Epley maneuver treatment (“BPPV” group), or due to non-vestibular cause, e.g., cardiovascular, neuromuscular, or psychogenic condition (“non-vestibular” group). Only after all the testing was complete and participants categorized were they referred for later treatment.

### Data Analysis

For sinusoidal data, cycles (a cycle equals one period) with quick phases (resetting eye movements that bring the eye back to center once they reach the edge of the oculomotor range) were removed, and the remaining (i.e., slow-phase vestibular eye movements) cycles were overlaid and averaged on a point-by-point basis. Gain and phase were computed using the horizontal eye and head velocities. Gain was defined as the eye/head quotient of amplitude for least-squares best-fit pure sinusoids approximating the eye and head velocity mean traces. Gain and phase were expressed with the convention that unity gain and zero phase imply a perfectly compensatory VOR; positive phase implies that eye movements lead head movements and negative phase implies that eye movements lag behind head movements. During testing at 1.6 and 2 Hz decoupling that sometimes occurred between the head and chair was detected by a large increase in phase shift and decrease in gain compared to the 1 Hz data. Decoupled data were not included in the analysis.

For unidirectional transient data (i.e., acceleration steps), the points in time where the absolute magnitude of eye velocity was maximal immediately after acceleration (P_EXC_, excitatory stimulus) and after de-acceleration (P_INH_, inhibitory stimulus) were determined and used to calculate the excitatory VOR gain (eye/head velocity at P_EXC_) and the inhibitory VOR gain (at P_INH_), respectively. Starting at time P_EXC_, points on the eye velocity trace immediately before the start, and after the end, of quick phases (therefore, only selecting the slow-phase data) were manually chosen and an exponential curve was fitted to the data in order to calculate the excitatory TC. TC was defined as the time taken for eye velocity to decay to 63% of peak velocity under constant velocity rotation. An unusually long (>30 s) vestibular TC suggests a central vestibular disorder (24). The inhibitory TC was similarly calculated by fitting points after time P_INH_. The OKN gain was calculated by selecting slow-phase eye velocity data points (i.e., excluding quick phase data points as per above) during the constant velocity rotation period with lights on. The average velocity of these points was calculated and divided by 50°/s (i.e., eye/head velocity) to calculate the OKN gain. The OKN TC was calculated by fitting an exponential curve to the slow-phase data during lights off, starting immediately after the period with lights on. The VOR and OKN, gains and TCs, calculations were identical for both leftward and rightward transient data.

### Statistical Analysis

Statistical analysis was performed using Matlab 2008a (Mathworks, USA) and Excel 2007 (Microsoft, USA) software. We used a mixed-design repeated measures analysis of variance (ANOVA) with two-, three-, and four-factor interactions to analyze the data (25). Main and interaction effects of participant vestibular rehabilitation group (*group*: “lesion,” “non-vestibular,” or “BPPV”), test type (*test*: “VVOR” or “VOR”), eye used to calculate the gain or phase (*eye*: “left,” “right”), and test frequency (*frequency*: 0.1, 0.2, 0.4, 0.5, 0.8, 1, 1.6, and 2 Hz) on sinusoidal VOR gain and phase were investigated. Main and interaction effects of “*group*,” “*eye*,” vestibular stimulus applied with respect to the lesion side (*sameside*: “yes” or “no”) and vestibular stimulus type (*stimulus*: “excitatory,” “inhibitory”) on transient VOR gain and TC were investigated. Main and interaction effects of “*group*,” “*eye*,” and “*sameside*” on OKN gain and TC were investigated. Main and interaction effects of “*group*,” “*sameside*,” and canal (*canal*: “horizontal,” “anterior,” and “posterior”) on DVA score were investigated. Main and interaction effects of “*group*” on total physical, total emotional, total functional, and grand total DHI scores were investigated. We report variables (or factors) with 95% confidence (i.e., 5% significance) as significant and those with 90% confidence (i.e., 5–10% significance) as trends. We also report the effect size η^2^ for ANOVA, which was considered as small 0.005–0.05, medium 0.05–0.125, or large >0.125; and effect size Cohen-*d* for *z-test*, which was considered as small 0.15–0.45, medium 0.45–0.75, or large >0.75 (26).

## Results

### Demographics

Ten participants (5 female, 5 male) were categorized into the lesion group, 19 (15 female, 4 male) into the BPPV group, and 47 (26 female, 21 male) into the non-vestibular group. There was no difference in mean ages between non-vestibular (68.3 ± 9.5 years), lesion (70 ± 11.8 years), and BPPV (70.1 ± 8.6 years) groups (ANOVA: “*group*” variable, *P* = 0.749, η^2^ = 0.008).

We categorized the last dizzy episode for each participant into six time periods [test day (prior to testing), last week, last month, last 3 months, last 6 months, last year]. For the lesion group, three participants experienced a dizzy episode on the day, four in the last week, and three in the last month. For the BPPV group, five on the day, nine in the last week, three in the last month, one in the last 3 months, and one in the last year. For the non-vestibular group, 12 on the day, 10 in the last week, 16 in the last month, 5 in the last 3 months, and 4 in the last 6 months.

For the 19 BPPV patients during Dix–Hallpike testing; 8 had upbeat right torsional nystagmus during right posterior canal testing only; 5 upbeat left torsional nystagmus during left posterior canal testing only; and 6 had upbeat with left torsional nystagmus during left posterior canal testing and upbeat with right torsional nystagmus during right posterior canal testing. Fourteen out of the 19 BPPV participants described head movement as a clear trigger for their dizziness, which was predominantly a spinning sensation.

### Clinical Head Impulse Test

The cHIT was performed in 68 out of the 76 participants. The remaining eight could not be tested due to difficulty with moving their head due to neck stiffness and or pain. The cHIT was positive in 8/42 in the non-vestibular group, 1/9 in the lesion group, and 4/17 in the BPPV group. There was no difference in these proportions between groups (*z*-test: lesion vs. non-vestibular, *P* = 0.569, Cohen-*d* = −0.159; BPPV vs. non-vestibular, *P* = 0.697. Cohen-*d* = 0.101; lesion vs. BPPV, *P* = 0.447, Cohen-*d* = 0.303).

### Head Thrust Dynamic Visual Acuity

Table 1 shows the mean htDVA scores for non-vestibular, lesion, and BPPV groups as well as the proportion of participants classified as normal, borderline, and abnormal. For presentation of non-vestibular group data in Table 1 only, ipsilesional is left and contralesional is right.

**Table 1:** **Summary of mean htDVA scores for non-vestibular, lesion (non-BPPV), and BPPV groups as well as the proportion of participants classified as normal, borderline, and abnormal (≤0.158, >0.158 and ≤0.316, and >0.316, respectively)**.

Side	Canal	Non-vestibular			Lesion (non-BPPV)			BPPV		
		Affected	No.	Mean ± SD	Affected	No.	Mean ± SD	Affected	No.	Mean ± SD
Ipsi.	Hor.	Normal	34/47	0.154 ± 0.131	Normal	3/15	0.300 ± 0.139	Normal	11/23	0.200 ± 0.131
		Borderline	8/47		Borderline	5/15		Borderline	9/23	
		Abnormal	5/47		Abnormal	7/15		Abnormal	3/23	
	Ant.	Normal	28/44	0.182 ± 0.180	Normal	5/15	0.237 ± 0.112	Normal	14/22	0.137 ± 0.065
		Borderline	8/44		Borderline	4/15		Borderline	8/22	
		Abnormal	8/44		Abnormal	6/15		Abnormal	0/22	
	Post.	Normal	18/40	0.189 ± 0.116	Normal	2/15	0.328 ± 0.151	Normal	5/20	0.249 ± 0.127
		Borderline	16/40		Borderline	5/15		Borderline	10/20	
		Abnormal	6/40		Abnormal	8/15		Abnormal	5/20	
Contra.	Hor.	Normal	31/45	0.160 ± 0.143	Normal	5/5	0.113 ± 0.041	Normal	8/13	0.211 ± 0.195
		Borderline	9/45		Borderline	0/5		Borderline	2/13	
		Abnormal	5/45		Abnormal	0/5		Abnormal	3/13	
	Ant.	Normal	20/43	0.208 ± 0.138	Normal	2/5	0.195 ± 0.081	Normal	8/12	0.135 ± 0.068
		Borderline	12/43		Borderline	2/5		Borderline	4/12	
		Abnormal	11/43		Abnormal	1/5		Abnormal	0/12	
	Post.	Normal	17/39	0.203 ± 0.115	Normal	3/5	0.245 ± 0.210	Normal	6/12	0.188 ± 0.096
		Borderline	15/39		Borderline	1/5		Borderline	4/12	
		Abnormal	7/39		Abnormal	1/5		Abnormal	2/12	

*For the non-vestibular group, ipsilesional is left and contralesional is right*.

The factors which affected the htDVA score were the canal tested (ANOVA: *canal*, *P* < 0.05, η^2^ = 0.015) and participant group (ANOVA: *group*, *P* < 0.01, η^2^ = 0.021). There was a close to 5% significant difference between ipsilesional and contralesional rotations (ANOVA: *sameside*, *P* = 0.0506, η^2^ = 0.008), and toward an interaction between *sameside* and *group* (ANOVA: *P* = 0.0715, η^2^ = 0.007).

Sub-analysis comparing only non-vestibular and lesion group data showed a significant difference in htDVA score between rotations toward the ipsilesional and contralesional (both left and right sides were pooled for the non-vestibular group) sides (ANOVA: *sameside* variable, *P* < 0.02, η^2^ = 0.019). Additionally, for the lesion group only, horizontal canal htDVA scores were significantly different between ipsilesional and contralesional sides (ANOVA: *sameside*, *P* < 0.01, η^2^ = 0.058).

Sub-analysis comparing only BPPV and lesion group data showed a significant difference in htDVA score between groups (ANOVA: *group*, *P* < 0.002, η^2^ = 0.058) as well as between canals (ANOVA: *canal*, *P* < 0.01, η^2^ = 0.068) and between ipsilesional and contralesional sides (ANOVA: *sameside*, *P* < 0.05, η^2^ = 0.023). There was a close to 5% significance interaction between group and lesion sides (ANOVA: *P* = 0.0558, η^2^ = 0.02). Examination of the canals individually indicated that anterior canal htDVA scores were significantly different between BPPV and lesion groups (ANOVA: *group*, *P* < 0.001, η^2^ = 0.217). Additionally, there was a trend toward a difference in posterior canal DVA scores between BPPV and lesion groups (ANOVA: *group*, *P* = 0.0726, η^2^ = 0.07) as well as between ipsilesional and contralesional sides (ANOVA: *sameside*, *P* = 0.0996, η^2^ = 0.059). There were no differences between non-vestibular and BPPV groups.

### Dizziness Handicap Inventory

Table 2 shows the mean DHI scores for physical, emotional, and functional parts of the questionnaire as well as the mean grand total DHI scores for the non-vestibular, lesion, and BPPV groups.

**Table 2:** **Summary of mean DHI scores for physical, emotional, and functional parts of the questionnaire as well as the mean total DHI scores for non-vestibular, lesion, and BPPV groups**.

DHI score	Non-vestibular (*n* = 47)	Lesion (non-BPPV) (*n* = 10)	BPPV (*n* = 19)
	Mean ± SD	Mean ± SD	Mean ± SD
Physical	10.1 ± 6.65	11.8 ± 6.76	11.4 ± 6.96
Emotional	4.85 ± 5.56	12.4 ± 8.42	6.42 ± 6.95
Functional	8.04 ± 7.48	13.2 ± 8.34	8.74 ± 8.17
Total	23.0 ± 16.5	37.4 ± 19.2	26.5 ± 18.8

*Total scores of ≤39, ≥40 and ≤69, and ≥70 indicate low, moderate, and severe self-perception of handicap, respectively*.

There was a significant difference in the total emotional score between groups (ANOVA: *group, P* < 0.01, η^2^ = 0.138). There was a close to 5% significant difference in grand total scores between groups (ANOVA: *group*, *P* = 0.0643, η^2^ = 0.072).

Sub-analysis comparing only lesion and non-vestibular group data showed a significant difference in the total emotional (ANOVA: *group, P* < 0.001, η^2^ = 0.186) and grand total scores (ANOVA: *group*, *P* < 0.02, η^2^ = 0.098) as well as a trend toward a difference in total functional scores (ANOVA: *group*, *P* = 0.0572, η^2^ = 0.064). There was a close to 5% significant difference in total emotional scores between lesion and BPPV groups (ANOVA: *group*, *P* = 0.0504, η^2^ = 0.135). There was no difference in DHI scores between the BPPV and non-vestibular groups.

### Sinusoidal Horizontal VOR and VVOR Testing

**Figure 1:**
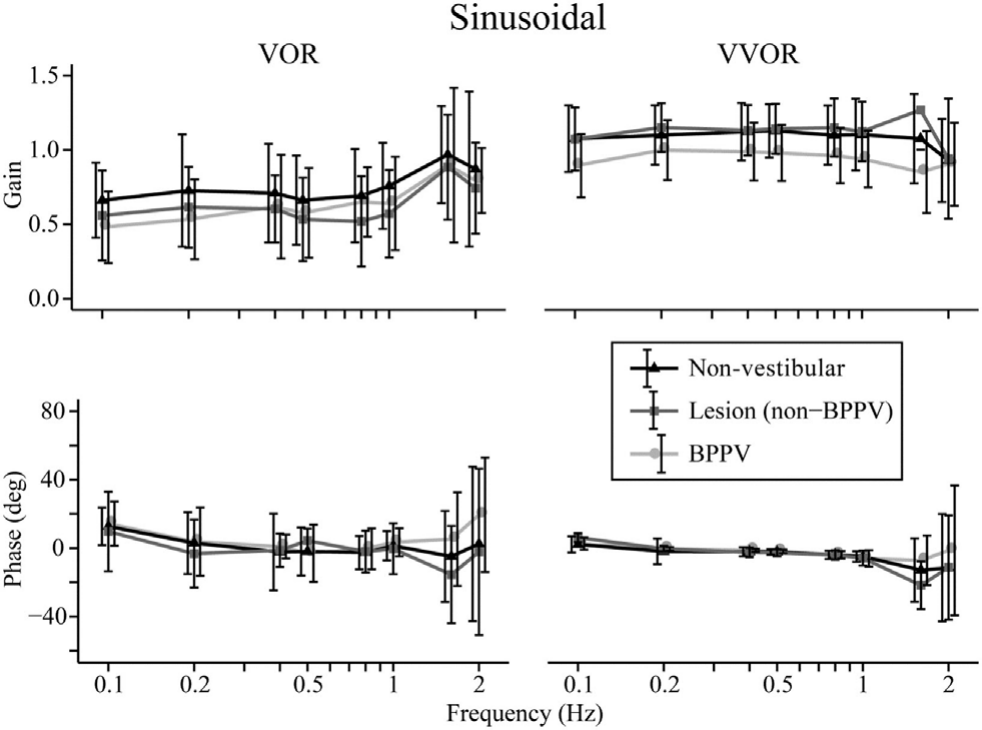
**Mean sinusoidal vestibulo-ocular reflex (VOR) and visual VOR (VVOR) gains and phases for non-vestibular, lesion, and BPPV groups over all frequencies**.

The factors that affected the gain were the test protocol (VOR or VVOR; ANOVA: *test, P* < 0.0001, η^2^ = 0.275), participant group (ANOVA: *P* < 0.0001, η^2^ = 0.026), and testing frequency (ANOVA: *P* < 0.0001, η^2^ = 0.017). There was a significant interaction between test protocol and frequency (ANOVA: *P* < 0.0001, η^2^ = 0.033). As shown in Figure 1, the VOR gain increases at frequencies ≥1.6 Hz (ANOVA: *frequency*, *P* < 0.0002, η^2^ = 0.025).

Sub-analysis comparing only lesion and non-vestibular group data showed the factors that affected gain were test protocol (ANOVA: *P* < 0.001, η^2^ = 0.297), group (ANOVA: *P* < 0.02, η^2^ = 0.003), and frequency (ANOVA: *P* < 0.001, η^2^ = 0.019) particularly between 0.5 and 1 Hz where the mean lesion VOR gain was ~25% lower than the mean non-vestibular VOR gain. Phase was affected by test protocol (ANOVA: *P* < 0.0001, η^2^ = 0.022) and frequency (ANOVA: *P* < 0.0001, η^2^ = 0.069), and there was a significant interaction between test protocol and frequency (ANOVA: *P* < 0.01, η^2^ = 0.013). The frequency significantly affected phase during VOR testing only when 0.1, 1.6, and 2 Hz were included in the analysis (when these frequencies were removed, frequency no longer became significant), whereas it significantly affected phase at all frequency ranges of VVOR testing (ANOVA: *P* < 0.0001, η^2^ = 0.156).

Sub-analysis comparing only BPPV and non-vestibular group data showed the factors that affected gain were test protocol (ANOVA: *P* < 0.001, η^2^ = 0.238), group (ANOVA: *P* < 0.001, η^2^ = 0.028), and frequency (ANOVA: *P* < 0.001, η^2^ = 0.015). Between 0.5 and 1 Hz, the mean BPPV VOR gain was ~13% lower than the mean non-vestibular VOR gain. Phase was affected by test protocol (ANOVA: *P* < 0.0001, η^2^ = 0.032), group (ANOVA: *P* < 0.01, η^2^ = 0.005), and frequency (ANOVA: *P* < 0.001, η^2^ = 0.045). There were significant interactions between test protocol and frequency (ANOVA: *P* < 0.0001, η^2^ = 0.022) as well as group and frequency (ANOVA: *P* < 0.002, η^2^ = 0.012). There was a VOR phase difference between BPPV and non-vestibular groups (ANOVA: *P* < 0.02, η^2^ = 0.007), which was not significant during testing at <2 Hz (*P* = 0.0871, η^2^ = 0.004). Frequency did not affect the VOR phase for frequencies >0.1 and <2 Hz. There was a difference between BPPV and non-vestibular groups for VVOR phase (ANOVA: *P* < 0.05, η^2^ = 0.005), which was no longer significant during testing at <2 Hz (*P* = 0.0627, η^2^ = 0.003). Frequency significantly affected the VVOR phase at all frequency ranges of VVOR testing (ANOVA: *P* < 0.0001, η^2^ = 0.085).

### Transient (Acceleration Steps) Horizontal VOR Testing

**Figure 2:**
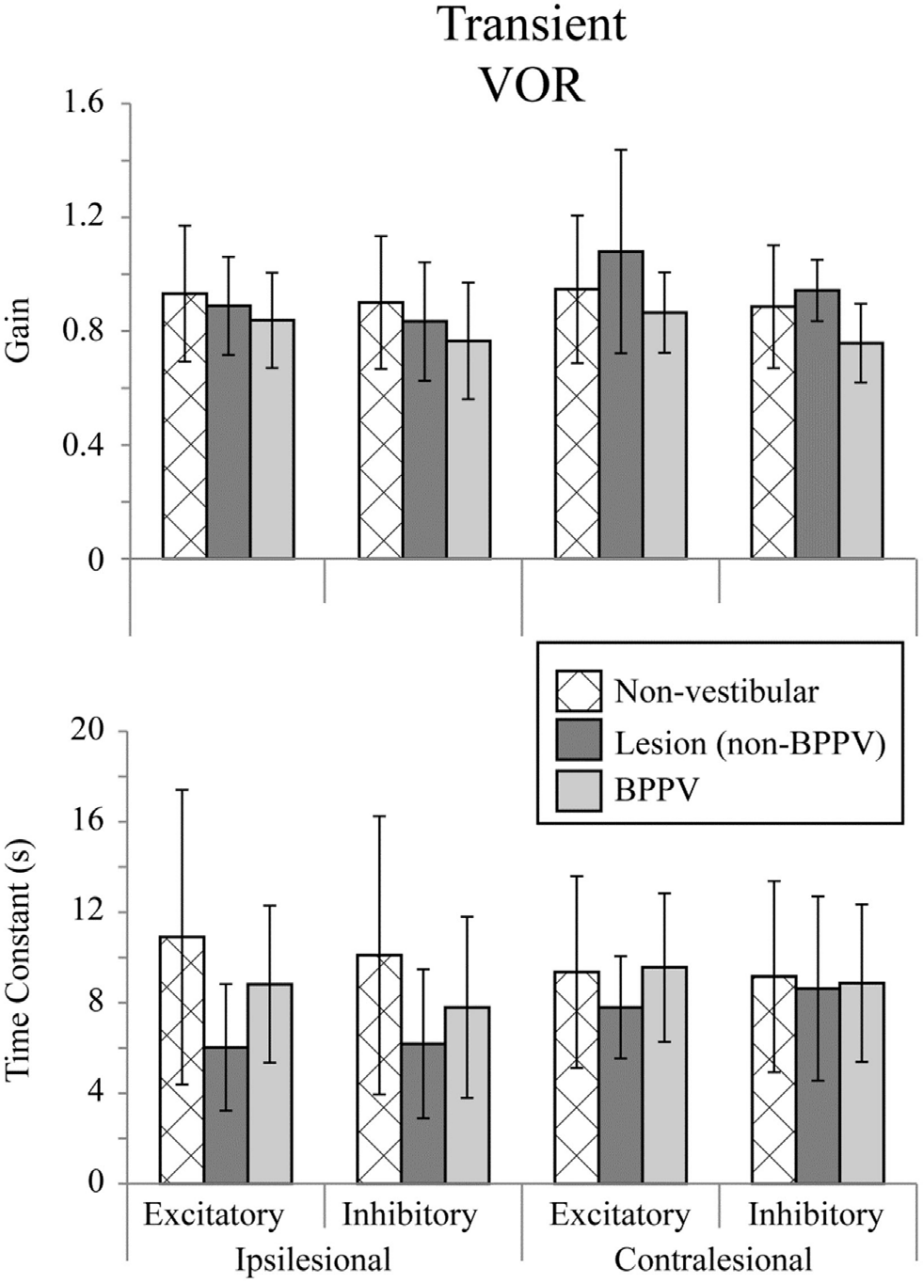
**Mean transient (acceleration step) VOR gains and time constants for ipsilesional and contralesional rotational stimuli that are excitatory (measured during acceleration) or inhibitory (measured during de-acceleration) for non-vestibular, lesion, and BPPV groups**. For the non-vestibular group, ipsilesional is left and contralesional is right.

**Figure 3:**
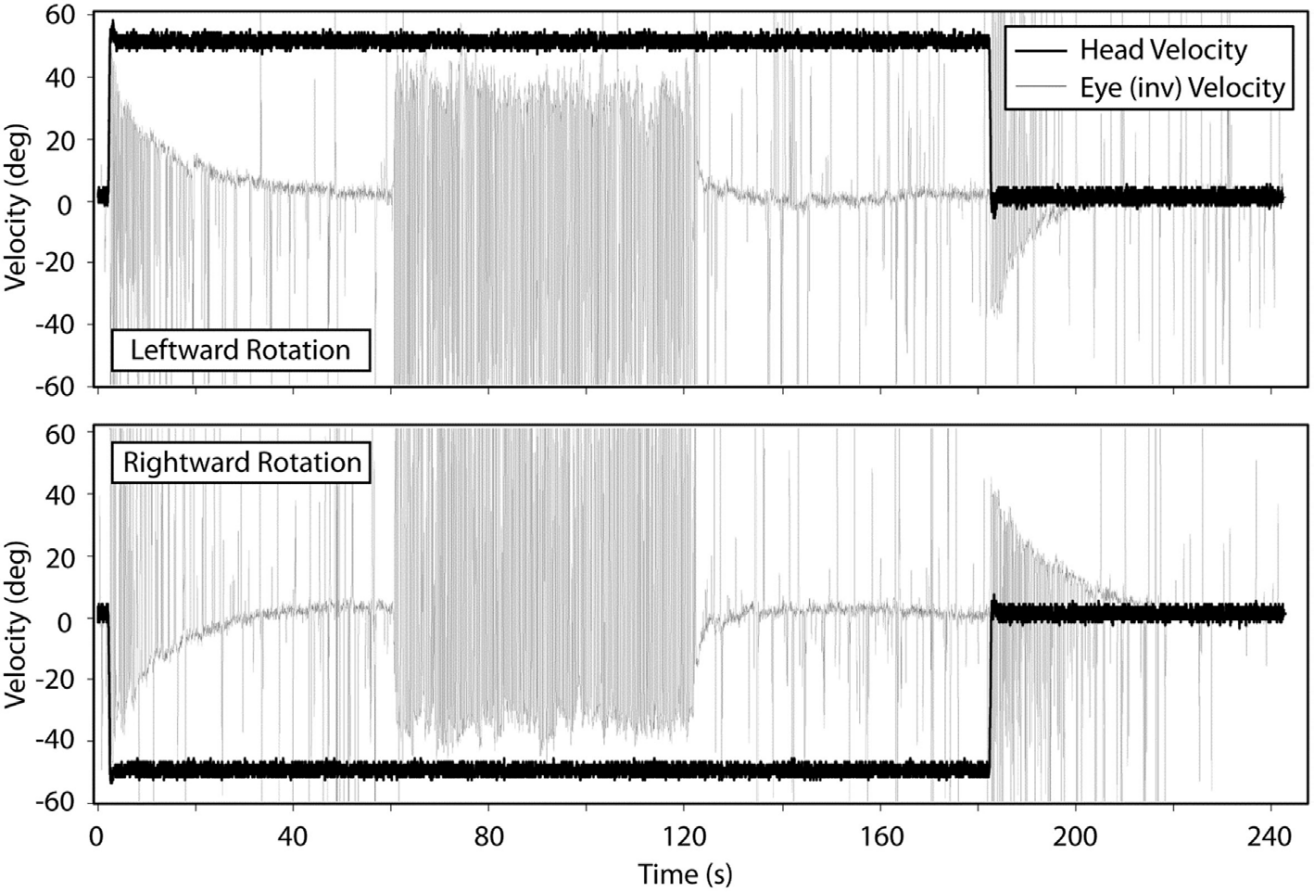
**Typical response from a participant in the non-vestibular group during transient VOR and OKN testing**. Top panel shows responses during leftward rotations and bottom panel for rightward rotations.

Figure 2 shows the means for VOR gain and TC for ipsilesional and contralesional transient unilateral rotations that were excitatory (during acceleration) and inhibitory (during de-acceleration) for the non-vestibular, lesion, and BPPV groups. For the non-vestibular group and Figure 2 only, ipsilesional is left and contralesional is right. Figure 3 shows the typical raw trace response in a participant from the non-vestibular group.

The factor which affected the acceleration step gain was whether the stimulus was excitatory or inhibitory (ANOVA: *stimulus*, *P* < 0.05, η^2^ = 0.019). There was a close to 5% significant effect of group on the acceleration step gain (ANOVA: *group*, *P* = 0.0624, η^2^ = 0.021) and TC (ANOVA: *group*, *P* = 0.0926, η^2^ = 0.018).

Sub-analysis comparing only non-vestibular and lesions group data showed a trend toward a difference in acceleration step gain between ipsilesional and contralesional sides (both left and right sides were pooled for the non-vestibular group) (ANOVA: *sameside*, *P* = 0.0798, η^2^ = 0.017). The acceleration step TC was not different (ANOVA: *group*, *P* = 0.651, η^2^ = 0.001) between lesion and non-vestibular groups. Within the lesion group, there was a trend toward a difference in TC between ipsilesional and contralesional rotations (ANOVA: *P* = 0.0809, η^2^ = 0.095).

Sub-analysis comparing only non-vestibular and BPPV group data showed a significant difference in acceleration step gain between groups (ANOVA: *P* < 0.05, η^2^ = 0.021), especially during inhibition stimulation (ANOVA: *P* < 0.05, η^2^ = 0.038). However, there were no significant factors which affected the TC.

Sub-analysis comparing only BPPV and lesion group data showed a significant difference in acceleration step gain between groups (ANOVA: *P* < 0.05, η^2^ = 0.053). For inhibitory stimuli, there was a trend toward a difference in acceleration step gain between BPPV and lesion groups (ANOVA: *P* = 0.0623, η^2^ = 0.069). There was also a significant difference in the TC between BPPV and lesion groups (ANOVA: *P* < 0.05, η^2^ = 0.06), as well as a trend toward a difference in TC between ipsilesional and contralesional rotations (ANOVA: *P* = 0.0796, η^2^ = 0.029). For excitatory stimuli, there was a difference in TC between BPPV and lesion groups (ANOVA: *P* < 0.02, η^2^ = 0.123).

**Figure 4:**
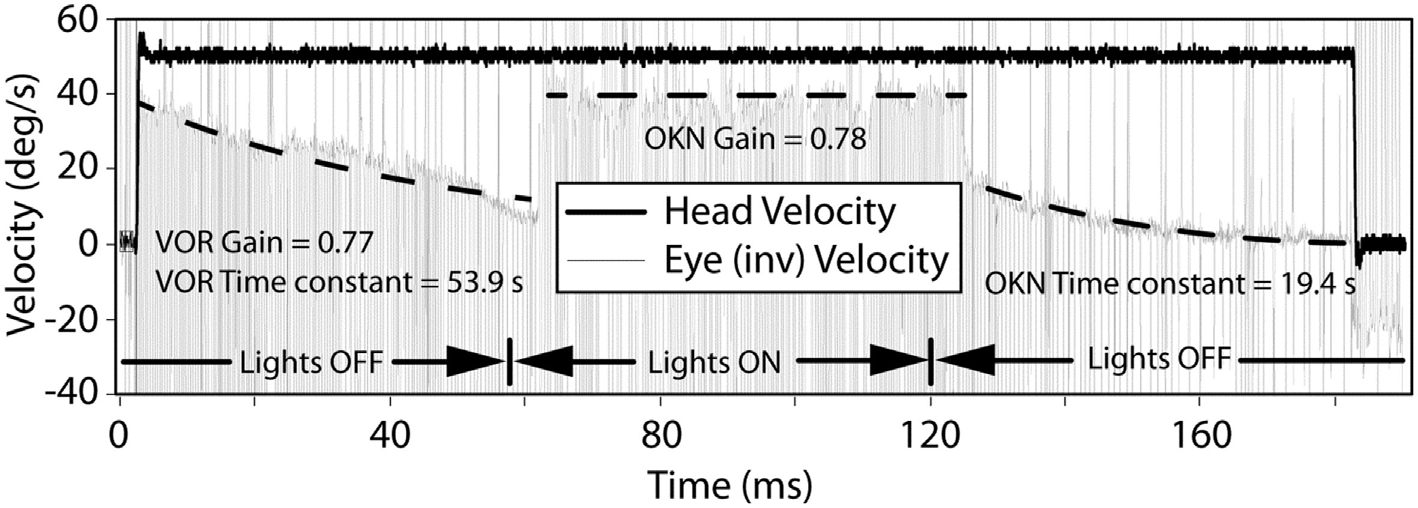
**The VOR and OKN response during leftward rotation in a participant with central vestibular dysfunction**. The left and right dashed lines indicate exponential decay fits to the slow-phase component of the eye movement in darkness and are used to calculate the VOR gain, VOR, and OKN time constants. The middle dashed line indicates a linear fit to the slow-phase component of the eye movement in light and is used to calculate the OKN gain.

Transient VOR testing identified one participant with excitatory response, displayed in Figure 4, with exponential decay longer than normal duration.

### Optokinetic Testing (OKN)

**Figure 5:**
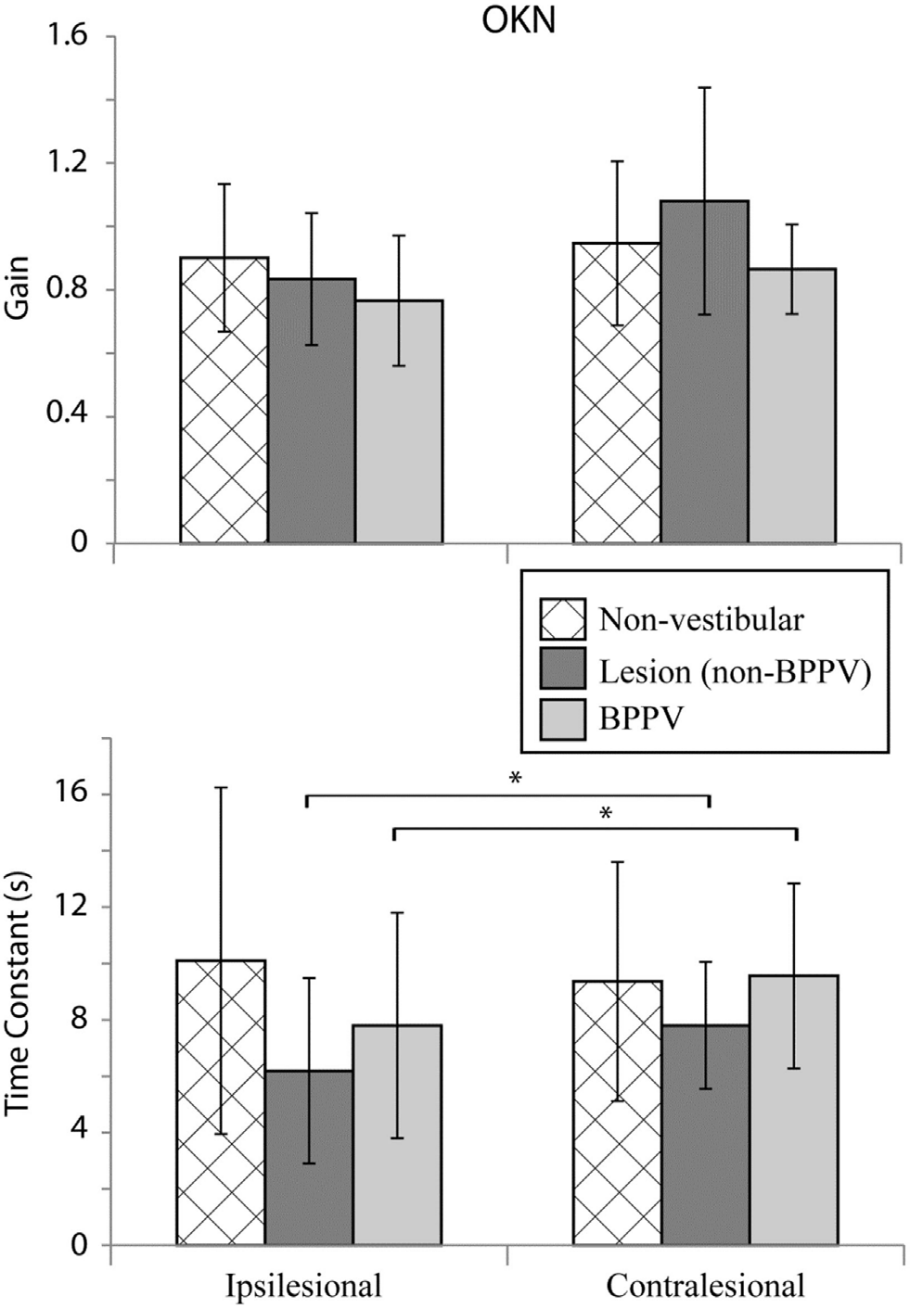
**Mean optokinetic nystagmus (OKN) gains and time constants for non-vestibular, lesion, and BPPV groups for ipsilesional and contralesional rotational stimuli**. For the non-vestibular group, ipsilesional is left and contralesional is right. The * denotes significant difference.

Figure 5 shows the mean values for optokinetic gain and TC for the non-vestibular, lesion, and BPPV groups during ipsilateral and contralateral stimuli. For the non-vestibular group and Figure 5 only, ipsilesional is left and contralesional is right.

The factor which affected the OKN TC was whether the stimulus was ipsilesional or contralesional (ANOVA: *sameside*, *P* < 0.05, η^2^ = 0.035). No factors affected the OKN gain. Within the lesion group, the TC was significantly different between ipsilesional and contralesional rotations (*P* < 0.01, η^2^ = 0.467).

## Discussion

Thirty-eight percent of participants had a detectable peripheral vestibular disorder (29/76) and 1% a central vestibular disorder (1/76), which was the likely cause of their dizziness. Of those with a vestibular cause, 63% (19/30) had BPPV, which is higher than the previously reported ~25% in dizziness clinic populations (18, 19). This 2.5 times larger prevalence can be explained by the fact that participants had experienced dizziness in the last year, which meant this population was biased toward having a vestibular disorder. However, it is also important to note that due to the difference in time between dizzy spell and assessment, for some of the participants, vestibular disorders may have resolved or progressed to chronic and well-compensated, which would have made detection less possible.

### Head Thrust Dynamic Visual Acuity

For the non-vestibular group, htDVA scores were similar between left and right sides, whereas in the lesion group, there was a difference between ipsilesional and contralesional sides. For the BPPV group, the posterior canal htDVA score on the ipsilesional side was highest (worst).

The difference in lesion group horizontal htDVA scores between ipsilesional and contralesional head rotations is due to loss of vestibular function, as has been previously reported [e.g., Ref. (17)]. However, for all groups, there was a trend toward a difference in htDVA scores depending on the canal tested, i.e., horizontal, anterior, or posterior. In fact, within BPPV and lesion groups, this difference was significant. This finding is understandable for the BPPV group given that BPPV commonly occurs in the posterior canals (as was the case in all our participants) due to their anatomical position. The relationship between posterior canal htDVA score and BPPV has not been previously reported. Our finding suggests that BPPV not only inappropriately persists a vestibular-evoked eye movement once the head has stopped rotating but also alters the VOR eye movement *during* the head movement (i.e., when the optotype is flashed) in a way that affects DVA. By contrast, for the lesion group, it is not clear why the posterior canal htDVA score was higher than the other canals when testing the contralesional side. During htDVA testing, it was noted that in our population ~59% (45/76) used bifocal or multifocal glasses. The vertical direction of the head thrusts during anterior and posterior canal testing is likely to have caused viewing lens crossover, which could have increased htDVA scores during the testing for those canals.

### Dizziness Handicap Inventory Scores

In examining the DHI scores, it appears BPPV has less effect on emotional and functional quality of life than other vestibular conditions. Participants with BBPV perceived their handicap to be somewhere between lesion and non-vestibular participants and the difference in total scores was only statistically different between the lesion and non-vestibular groups.

### Sinusoidal Horizontal VOR and VVOR

We found significant differences in gain and phase between BPPV and non-vestibular groups. Manifestation of BPPV usually occurs in the posterior canals, given their anatomical position, so presumably the horizontal VOR should not be affected by BPPV, yet our results suggest it is. Our findings show that the BPPV group sinusoidal horizontal VOR gain was between the lesion and non-vestibular group gains, especially between 0.5 and 1 Hz, which seems to be the critical point for detecting vestibular disorder during human rotary chair testing. Presumably, this is because the VOR contributes to vision stabilization starting at around 0.5 Hz. During higher-frequency chair rotations, decoupling can occur between the head and chair resulting in larger response variation and decreased sensitivity. Across all groups, the VOR gain increased at stimulus frequencies ≥1.6 Hz, whereas VVOR gain decreased at 2 Hz, which agrees with the findings of Li et al. (11) that suggested decreasing gain with increasing frequency. Our VOR phase analysis showed no significant difference between non-vestibular and lesion groups, whereas at higher frequencies (≥1.6 Hz), there were differences between non-vestibular and BPPV as well as BPPV and lesion groups.

### Transient (Acceleration Steps) Horizontal VOR

With Transient VOR testing, differences were noted primarily between BPPV compared to non-vestibular and lesion groups, respectively. There was no difference in TC between non-vestibular and BPPV groups, whereas gain was shown to differ, especially during inhibitory stimulation. This finding is consistent with our sinusoidal VOR data, which also showed a difference between BPPV and non-vestibular groups. The largest (albeit not statistically significant) difference between ipsilesional vs. contralesional gain was observed in the lesion group during excitatory stimulation. Similarly, the largest difference between ipsilesional and contralesional TC was observed in the lesion group during inhibitory stimulation. Transient VOR testing identified one participant with likely central vestibular dysfunction.

### Optokinetic Nystagmus

With regard to horizontal OKN testing, for the lesion and BPPV groups, there was a significant difference in TC between ipsilesional and contralesional rotations. No other significant results were obtained from any of the comparisons for TC or gain. This is a somewhat surprising result given that the OKN response is a mixture of optokinetic and smooth pursuit systems that should perfectly stabilize vision during 50°/s constant velocity head rotations in the light (24). A decrease in OKN gain or increase in TC would be indicative of central, most likely cerebellar, injury. In fact, in the case of the one participant with central vestibular dysfunction described above (see Results and Figure 4), the OKN TC was almost seven times the normal TC of about 3 s.

### Technical Issues

There were some issues with the amount of viable data we were able to collect, especially with regard to transient VOR testing and OKN testing. This happened due to some participants blinking frequently (resulting in noisy data) and some participants closing their eyes during OKN testing to reduce nystagmus (leading to a lack of reinforcement of the reflex and consequently a lack of OKN response). During sinusoidal VOR testing, despite tight fitting head and body restraints and straps, decoupling between the head and chair, especially in larger participants, was unavoidable at high frequencies. This limitation reduced the amount of data included at these frequencies.

## Conclusion

Overall, the results suggest that htDVA, DHI, sinusoidal VOR (particularly at 1 Hz), transient VOR, and OKN testing are all useful tools for detecting peripheral vestibular causes of dizziness in older people. Our most surprising finding was that BPPV and lesion groups had similarly low gains compared to the non-vestibular group during sinusoidal horizontal VOR testing. We also observed a possible relationship between BPPV and an isolated increase in the affected posterior canal htDVA score, which warrants further investigation.

## Author Contributions

AC collected DVA and rotary chair data, wrote thesis upon which this manuscript was drafted. JM helped conceive the study, recruited participants, and collected DHI, BPPV, and cHIT data, proofread manuscript. PH collected DVA and rotary chair data, wrote the processing data software. SL helped conceive the study, obtained ethics approval, and proofread the manuscript. AM helped conceive the study, analyzed (statistical, summary, and figures) the data, and wrote the draft and final manuscripts.

## Conflict of Interest Statement

The authors declare that the research was conducted in the absence of any commercial or financial relationships that could be construed as a potential conflict of interest.
